# Contributing Reviewers in 2021

**DOI:** 10.1055/s-0042-1742488

**Published:** 2022-02-11

*Global Medical Genetics* wishes to recognize those who contributed as an expert peer-reviewer of submitted scientific papers in 2021.

Thank you for your contributions to *Global Medical Genetics* .

Iwona Żur

Tomer Ziv-Baran

Andrea Zini

Qian-Hao Zhu

Jiang Zhou

Yingmei Zhang

Xinjiang Zhang

Ángel Zarain-Herzberg

Sarah E. Zanders

Hakimeh Zali

Raymond Yung

Yonggui Yuan

Jin-qing Yuan

Hao Yu

Chao Yu

Pierre Youinou

Yeliz Yol

Va yo

T. Ylisaukko-oja

Hiroyuki Yasuda

Bing Yao

Junichi Yamaguchi

Suowen Xu

Claudia Wylezich

Yah-Huei Wu-Chou

Wei-Te Wu

Jianghong Wu

Jiande Wu

Kris C. Wood

Hye Ryun Woo

Jongkonnee Wongpiyabovorn

Loo Keat Wei

Jill Wegrzyn

Milind Watve

Zehua Wang

Xiaojuan Wang

Weiping Wang

Ioannis A. Voutsadakis

Vandana Vinayak

Rafael Velazquez-Cruz

AB Vedamurthy

Sarinnapha M. Vasunilashorn

Sirisha Vammi

Zehra Oya Uyguner

Asta Tvarijonaviciute

Dilsad Turkdogan

Pınar Tulay

Ageliki Tsagaratou

Anthony Trewavas

Van Khanh Tran

Ann-Marie Torregrossa

Hirofumi Tomita

Lucia Tombolan

Pascale Tomasini

Maria Tomas

Pavel Tomancak

A. Tirella

Pilar S. Testillano

Leroy ten Dam

Duygu Tekin

Eleni Tani

Yuki Tamura

Murtaza M. Tambuwala

Gerasimos P. Sykiotis

Z. Renee Sung

Li-Peng Sun

Aihua Sun

Takaaki Sugiki

Irna Sufiawati

Jose Suazo

wenling SU

Chun-Li Su

Maja Stojiljkovic

Agnieszka Stembalska

Elizabeth K. Speliotes

Kayal DM Smita

Robert Smigiel

Michael K. Skinner

Sanjay M. Sisodiya

David Simar

Byoung-Soo Shin

Jin-Yuan Shih

Takahiko Shibahara

pei Shi

Brian M. Shewchuk

Lisha Shen

Pil Joon Seo

Aida Semic-Jusufagic

Barry Scott

Robert J. Schmitz

Mari Sandell

Maria, de la Paz Sanchez

Emine Turkmen Samdanci

Lynn Y. Sakai

Ozlem Saglam

Reza Safaralizadeh

Enrique Saez

Amets Saenz

Alexandre Rouen

Veronique Rossi

H. Llewelyn Roderick

Kate E. Riley

Bergmann M. Ribeiro

Silvia R. A. Reis

Theodore P. Rasmussen

Pragna Rao

Ana Lucia Carrinho Ayroza Rangel

Bhama Ramkhelawon

Elizabeth J. Radford

Larysa Y. Pylyp

Alkes L. Price

Shikha Prasad

Pranav Kumar Prabhakar

N. Pop-Jordanova

Lydia Poole

S. R. Phadke

Piero Perucca

A. M. Persico

Pascale Perrin

Israel Perez-Torres

A. Pena-Rosado

A. Pellicer

Ales Pecinka

Smaranika Pattnaik

Alireza Pasdar

Alireza Pasdar

Seppo Parkkila

Humberto, Jr. Parada

Pier Francesco Palamara

Bulent Ozpolat

Juan M. Orduna

M. Olive

Anna B. Ohlsson

B. Nowicka

Parisa Norouzitallab

Zahra Noormohammadi

Takahiro Nobuzane

Masha Y. Niv

Duc Hinh Nguyen

Yun Fong Ngeow

Praveen Kumar Neela

Hashem Nayeri

Satoshi Narumi

Masataka Narukawa

juan Nan

Gerson Nakazato

A. J. Munoz

Mridul Mukherji

Srabani Mukherjee

Hirohito Miura

Milica Miljkovic

Chiara Milani

L. Migliore

José Luis Micol

James J. Mezhir

Peter Meyer

Lisa Methven

Jan A. Mennigen

Shaban Mel

I.M. Medina-Díaz

Katsuhiro Masago

Efrén Martínez-Quintana

Marcella Martinelli

Lucia Margari

Chao Mao

Gaia Chiara Mannino

Licinio Manco

José E. Manautou

Kristiina Mäkinen

Abdolkarim Mahrooz

Magdy M. Mahfouz

Abbas Ali Mahdi

Asri Maharani

Paul S. Maddox

Mike Mackness

Renato Assis Machado

Malou A. Lugthart

Vito Longo

Hanns Lochmuller

Xian-zhi Liu

Shoufeng LIU

Charlotte Ling

Jinpiao Lin

Anatoly V. Lichtenstein

Marc Libault

Wilson Liao

Evi Lianidou

Xiaomin;Xiaowan Li;Li

xiaomin;juan li;he

Xiajun Li

Anna-Liisa Levonen

Tomoko Lee

Seung Jin Lee

Seong-Wook Lee

Jefferson LEE

M. A. Lebedeva

Douglas A. Lauffenburger

Junko Kusumi

Setor K. Kunutsor

Alexei P. Kudin

Ksenia V. Krasileva

Claudia Köhler

Do-Hyung Kim

Andre Salim Khayat

Naim A. Khan

Dr. Mahamad Irfanulla Khan

Golsa Ketabchi

Mohammad Amin Kerachian

Kathleen L. Keller

Fuminori Kawabata

Fuminori Kawabata

Ushang V. Kate

Jason Karamchandani

Ines Kapferer-Seebacher

Emine Kandemis

Rasime Kalkan

Perla Kaliman

Youn Joung Cho

Bryan W. Jones

Feras J. Jirjees

Emilio Jirillo

Chutima Jirapinyo

Junhyun Jeon

Musharraf Jelani

Ajit Jaiswal

Mayumi Iwasaki

Shinsuke Ito

Shumpei Ishikawa

Rachel Helen Horton

Nele Horemans

Ki Ho Hong

Georges Herbein

John E. Hayes

Ruilian Han

Agnieszka Halas

Kevin M. Haigis

Sanna Hagman

Zsuzsanna Gurdan

Pramod Kumar Gupta

A. Gugliucci

Alicja E. Grzegorzewska

Daniel Grimanelli

Stanislas Grassin-Delyle

Stephanie Graser

Isabel Gonçalves

Ricardo Santiago Gomez

J. Gohlke

Shamila Ginige

Tapash Chandra Ghosh

Romain Gherardi

Meenu Ghai

David P. Gavin

Brandon S. Gaut

Amparo Garcia-Tejedor

Philippe Gallusci

Alexandre Gagnon

Carlo Gaetano

Attila Frigyesi

Jennifer L. Freeman

Molly Fox

Natalia Forgacova

Antonino Forabosco

Eugenia Flores-Alfaro

Alberto Fernandez-Jaen

hongjuan Fang

Ligia Carla Faccin-Galhardi

William Evans

Won Sik Eum

Ebru Erzurumluoglu

Thomas Eggermann

Debasree Dutta

Burak Durmaz

Jorge Domínguez-Andrés

Korcan Demir

Clelia De-la-Peña

Frank de Vries

Maria Corazon A. De Ungria

Delcides Ferreira De Paula Junior

Daniel A. de Luis

Raul de Lucas

Jeremy J. Day

Aparup Das

Dr Amita Coutinho

Ricardo D. Coletta

Amander T. Clark

Stephan Claes

Thomas A. Ciulla

Fabrizio Citarella

Luisa Cimmino

Er-Chieh Cho

Maria Sole Chimenti

Z. Jeffrey Chen

Z. Jeffrey Chen

Pao-Yang Chen

Fuxue Chen

Prashen Chelikani

Pattama Chailertvanitkul

Carlo Cervellati

Clarissa Catale

Valeria Carola

Inmaculada Campos-Galindo

Bert Callewaert

Matteo Busconi

Russell J. Buono

Steven Buechler

Regina Maria Bringel Martins

Jason H. Brickner

J. Brezinova

Alison C. Brewer

An Boudewyns

Yvonne Böttcher

G. Borgonovo

Penelope E. Bonnen

Luca Reggiani Bonetti

Dragana Bogicevic

Peyman Björklund

Andriy Bilichak

Maitree Bhattacharyya

Iwona Ben-Skowronek

Moussa Benhamed

Akeila Bellahcène

Maik Behrens

Mohamed A. Bedaiwy

Diane M. Beckles

Claude Becker

Daniela Sanchez Bassères

Manish Bansal

Istvan Balogh

Alma Balestrazzi

Adayabalam S. Balajee

Bahman Bahramnejad

Melinda R. Baerwald

Fabian Baertling

Anwar Baban

Diana A. Averill-Bates

D. M. Avdjieva-Tzavella

Kapil K. Avasthi

Eliandro Reis Tavares

Emine Ikbal Atlı

Naureen Aslam

S. Aounzou

Gregory S. Antonarakis

Norma Almaraz-Abarca

Watfa Al-Mamari

Ahad Alizadeh

Mohammad Yousef Alikhani

Mercedes Alfonso-Prieto

İbrahim Akalin

Annabel Ahuriri-Driscoll

Gholamreza Ahmadian

Wasim Ahmad

Sarita Agarwal

Oladele Vincent Adeniyi

Ravinder Abrol

Francisco Abad-Santos

Nurul Syakima Ab Mutalib

